# Synthesis and Antioxidant Activity Evaluation of New Compounds from Hydrazinecarbothioamide and 1,2,4-Triazole Class Containing Diarylsulfone and 2,4-Difluorophenyl Moieties

**DOI:** 10.3390/ijms150610908

**Published:** 2014-06-17

**Authors:** Stefania-Felicia Barbuceanu, Diana Carolina Ilies, Gabriel Saramet, Valentina Uivarosi, Constantin Draghici, Valeria Radulescu

**Affiliations:** 1 Organic Chemistry Department, Faculty of Pharmacy, “Carol Davila” University of Medicine and Pharmacy, 6 Traian Vuia, 020956 Bucharest, Romania; E-Mails: ilies_diana@hotmail.com (D.C.I.); valeria_radulescu@yahoo.com (V.R.); 2 Pharmaceutical Technology Department, Faculty of Pharmacy, “Carol Davila” University of Medicine and Pharmacy, 6 Traian Vuia, 020956 Bucharest, Romania; E-Mail: gsaramet@gmail.com; 3 General and Inorganic Chemistry Department, Faculty of Pharmacy, “Carol Davila” University of Medicine and Pharmacy, 6 Traian Vuia, 020956 Bucharest, Romania; E-Mail: valentina_uivarosi@yahoo.com; 4 “C.D. Nenitescu” Institute of Organic Chemistry, Romanian Academy, 202B Splaiul Independenţei, 060023 Bucharest, Romania; E-Mail: cst_drag@yahoo.com

**Keywords:** hydrazinecarbothioamide, 1,2,4-triazole-3-thione, cyclization, alkylation, antioxidant activity, diarylsulfone, 2,4-difluorophenyl moiety

## Abstract

In the present investigation, new hydrazinecarbothioamides **4**–**6** were synthesized by reaction of 4-(4-X-phenylsulfonyl)benzoic acids hydrazides (X= H, Cl, Br) **1**–**3** with 2,4-difluorophenyl isothiocyanate and further these were treated with sodium hydroxide to obtain 1,2,4-triazole-3-thione derivatives **7**–**9**. The reaction of **7**–**9** with α-halogenated ketones, in basic media, afforded new S-alkylated derivatives **10**–**15**. The structures of the synthesized compounds have been established on the basis of ^1^H-NMR, ^13^C-NMR, IR, mass spectral studies and elemental analysis. The antioxidant activity of all compounds has been screened. Hydrazinecarbothioamides **4**–**6** showed excellent antioxidant activity and 1,2,4-triazole-3-thiones **7**–**9** showed good antioxidant activity using the DPPH method.

## 1. Introduction

Oxidation processes are intrinsic to the energy management of all living organisms and are therefore kept under strict control by several cellular mechanisms [1].

Free radicals are molecules, ions or atoms with unpaired electrons in their outermost shell of electrons [2]. These species, which are constantly formed in human body, can become toxic when generated in excess or in the presence of a deficiency in the naturally occurring antioxidant defenses. High levels of free radicals can cause damage to biomolecules such as lipids, proteins, enzymes and DNA in cells and tissues. This may result in many diseases such as: cancer, diabetes, cardiovascular and autoimmune diseases, and neurodegenerative disorders, aging, and other diseases through the violent reactivity of the free radicals [3,4,5].

Antioxidants are important compounds that reduce or neutralize the free radicals, thus protecting the cells from oxidative injury [6]. Therefore, considerable research has been directed towards the identification of new antioxidants to prevent radical-induced damage.

Over the years triazoles have become an important class of heterocyclic compounds in organic synthesis due to their various biological properties. It is well known that 1,2,4-triazole derivatives have therapeutic applications. Thus, there are various drugs incorporating in their structure the 1,2,4-triazole ring used as antifungal [7,8,9], antiviral [10] agents, aromatase inhibitors [11], *etc.* Among the 1,2,4-triazole derivatives, the mercapto- and the thione-substituted 1,2,4-triazole ring systems have been studied and so far a variety of biological properties have been reported for a large number of these compounds including antioxidant [12,13,14], antibacterial, antifungal [12,15,16,17,18], anticancer [17,19], hypolipidemic [20], anti-inflammatory [21] activity. Moreover, various S-alkylated 1,2,4-triazole-3-thiones showed antibacterial [22], antifungal [18,22], anti-inflammatory [23], and hypolipidemic [20] activities.

It has been reported that structural properties of triazoles, like moderate dipole character, hydrogen bonding capability, rigidity and stability under *in vivo* conditions are the main reasons for their superior pharmacological activities [24].

Many synthetic procedures exist for the synthesis of substituted 1,2,4-triazole-3-thiones. However, the development of simple, facile and efficient methodologies to get five-membered heterocycles is one of the major aspects in organic synthesis. Hydrazinecarbothioamides are valuable intermediates in a variety of synthetic transformations and useful as building blocks in the synthesis of biologically active heterocycles including synthesis of 1,2,4-triazole-thiones. In addition, hydrazinecarbothioamides derivatives exhibit various biological properties such as antioxidant [13,14,25,26], antibacterial [27], and antimycobacterial [28]*.*

Moreover, sulfone derivatives provide examples of an important class of bioactive compounds with biological activities including antibacterial, and anti-HIV-1 [29,30].

On the other hand, incorporation of one or several fluorine atoms into an organic molecule can enhance their biological potency, bioavailability, metabolic stability and lipophilicity. Enhanced lipophilicity may lead to easier absorption and transportation of molecules within biological systems [31].

Considering these published data and as a sequel to our research on the design and synthesis of biologically active new heterocycles from the triazole class [32,33,34,35], it was thought worthwhile to synthesize the novel title compounds and to evaluate them for their antioxidant activity.

In this study, we present the design, synthesis, characterization and evaluation of the antioxidant activity of the new hydrazinecarbothioamides, 1,2,4-triazole-3-thiones and some S-alkylated 1,2,4-triazole derivatives incorporating in their molecule diarylsulfone and 2,4-difluorophenyl moieties.

## 2. Results and Discussion

### 2.1. Chemistry

**Scheme 1:**
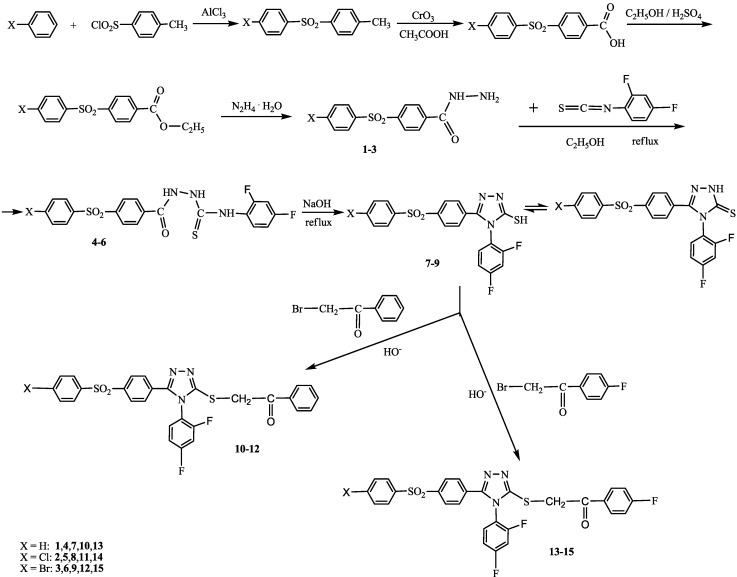
Synthetic route of the title compounds.

The reaction sequences employed for synthesis of title compounds are showed in Scheme 1. In the present work, *2-(4-(4-X-phenylsulfonyl)benzoyl)-N-(2,4-difluorophenyl)hydrazinecarbo-thioamides* **4**–**6** were synthesized by reaction of *4-(4-X-phenylsulfonyl)benzoic acid hydrazides* **1**–**3** (X = H, Cl, Br) with 2,4-difluorophenyl isothiocyanate, in absolute ethanol, at reflux. The 4-(4-X-phenylsulfonyl)benzoic acid hydrazides precursors **1**–**3** were prepared starting from Friedel-Crafts reaction of benzene or halobenzene with *p*-tosyl chloride, according to a previously reported method [36,37]. The hydrazinecarbothioamides **4**–**6** were refluxed in 8% sodium hydroxide solution to obtain *5-(4-(4-X-phenylsulfonyl)phenyl)-4-(2,4-difluorophenyl)-2H-1,2,4-triazole-3(4H)-thiones* **7**–**9** in equilibrium with thiole tautomer. The treatment of 1,2,4-triazoles **7**–**9** with α-halogenated ketones (2-bromoacetophenone or 2-bromo-4′-fluoroacetophenone), in basic media, produced the new S-alkylated 1,2,4-triazoles namely (*2-(5-(4-(4-X-phenylsulfonyl)phenyl)-4-(2,4-difluorophenyl)-4H-1,2,4-triazol-3-ylthio)-1-(phenyl/4-fluorophenyl)ethanones* **10**–**15** and not *N*-alkylated derivatives.

The structures of all synthesized compounds **4**–**15** were proven by ^1^H-NMR, ^13^C-NMR, MS spectra and elemental analysis.

The IR spectra of hydrazinecarbothioamide derivatives **4**–**6** exhibit a new absorption band at 1243–1258 cm^−1^ corresponding to C=S stretching vibration which confirms the nucleophilic addition reaction of 4-(4-X-phenylsulfonyl)benzoic acid hydrazides **1**–**3** to 2,4-difluorophenyl isothiocyanate. Also, in the IR spectra of these compounds **4**–**6** was presented as a strong characteristic absorption band for carbonyl group at 1663–1682 cm^−1^. The stretching bands corresponding to NH groups were observed in range 3150–3319 cm^−1^. In the IR spectra of compounds **7**–**9** no absorption band was detected about 1663–1682 cm^−1^ indicating the absence of C=O group of hydrazinecarbothioamides **4**–**6** which is evidence for the conversion of these compounds to 1,2,4-triazoles. Compounds **7**–**9** can exist in two tautomeric forms, *5-(4-(4-X-phenylsulfonyl)phenyl)-4-(2,4-difluorophenyl)-4H-1,2,4-triazole-3-thioles* and *5-(4-(4-X-phenylsulfonyl)phenyl)-4-(2,4-difluorophenyl)-2H-1,2,4-triazole-3(4H)-thiones* **7**–**9**. The spectral analysis (IR, ^1^H-NMR, ^13^C-NMR) shows that these compounds exist in the latter tautomeric form. Thus, in the IR spectra, the νS-H vibration band (~2500–2600 cm^−1^) was absent and the νC=S vibration band was observed in region 1247–1255 cm^−1^. Also, the presence of the νNH absorption band in 3278–3414 cm^−1^ region is an additional proof for the thione tautomeric form [13,38,39]. The structure of compounds **10**–**12** and **13**–**15** obtained by alkylation of triazoles **7**–**9** with α-halogenated ketones was confirmed by the presence in their IR spectra of a new strong stretching band in a 1678–1703 cm^−1^ region characteristic to C=O group. Also, new bands appeared in 2920–2965 cm^−1^region due to the presence of methylene group (νCH_2_). The disappearance of C=S stretching band in IR spectra supported the S-alkylation leading to the formation of compounds **10**–**15**.

Reaction of hydrazides **1**–**3** with 2,4-difluorophenyl isothiocyanate has been proven in ^1^H-NMR spectra of compounds **4**–**6** by the presence of three singlet signals at ~9.62, ~10.04 and ~10.90 ppm assigned to protons from three NH groups. Intramolecular cyclization of hydrazinecarbothioamides was confirmed in ^1^H-NMR spectra of compounds **7**–**9** by presence of a unique singlet at 14.42–14.48 ppm which can be attributed of NH proton from 1,2,4-triazol-3-thione nucleus [38]. The ^1^H-NMR spectra of all alkylated triazoles **10**–**15** displayed a singlet signal at δ = 4.99 (for **13**–**15**) and 5.02 ppm (**10**–**12**) assignable to S-CH_2_ protons. The absence of the signal due to NH in ^1^H-NMR spectra of compounds **10**–**15** and the presence of a new singlet for S-CH_2_ confirmed that 1,2,4-triazole-3-thiones **7**–**9** were converted into alkylated derivatives (**10**–**15**) in the reaction with α-halogenated ketones.

The ^13^C-NMR spectra of hydrazinecarbothioamides **4**–**6** showed two important signals characteristic of carbon atoms from C=O and C=S groups at δ 164.71–164.75 and 182.47 ppm, respectively [40]. In ^13^C-NMR of compounds **7**–**9** the C-3 and C-5 heterocyclic carbon from triazole nucleus resonated at 169.52–169.54 and 149.01–149.03 ppm, respectively. The signal of the C-3 quaternary carbon atom at ~169 ppm is characteristic of C=S group [12,13,41,42,43] which indicates the presence of the thione tautomeric form in solution. The most significant proof of the alkylation of triazoles **7**–**9** with 2-bromoacetophenone or 2-bromo-4'-fluoroacetophenone was the presence in ^13^C-NMR spectra of compounds **10**–**12** and **13**–**15** of two new signals at 191.42–192.73 and 40.33–40.46 ppm corresponding to C=O and S-CH_2_ carbon atoms from a phenacyl/4-fluorophenacyl group. In addition, the formation of S-alkylated and not of *N*-alkylated products was confirmed by the absence of a C=S characteristic peak at ~169 ppm in ^13^C-NMR spectra of **10**–**15**. The C-3 and C-5 heterocyclic carbons from these alkylated compounds resonate at 153.09–153.16 ppm (more shielded than the C-3 heterocyclic carbon from 1,2,4-triazoles **7**–**9**) and 153.01–153.09 ppm, respectively [12,43,44].

Moreover, the signals present in the NMR spectra corresponding to aromatic protons and carbons from 2,4-difluorophenyl-, 4-fluorophenyl- and 5-(4-(4-X-phenylsulfonyl)phenyl)*-*fragments prove the structure of the synthesized compounds. Further confirmations of the structure of the compounds were carried out by mass spectrometry and microanalysis (see experimental part).

### 2.2. Antioxidant Activity

The free radical scavenging activity of all compounds **4**–**15** was carried out in the presence of the stable free radical (1,1-diphenyl-2-picrylhydrazyl) DPPH using ascorbic acid (AA), *tert*-butyl-4-hydroxyanisole (BHA) and 2,6-bis(1,1-dimethylethyl)-4-methylphenol (BHT) antioxidant agents as positive control.

Although a number of methods are available for determination of the antioxidant activity, the DPPH method is very common, rapid and has been shown to be one of the most appropriate methods [12,45].

The DPPH solution has a deep purple color, with a strong absoption at 517 nm, and turns to yellow in the presence of antioxidants, which neutralizes the free radicals by pairing the DPPH odd electron with a hydrogen atom or by electron donation. Reduction of DPPH absorption at 517 nm represents the capacity of antioxidants to scavenge free radical [46].

The inhibitory effects of different concentrations of synthesized compounds on DPPH radical are presented in Table 1 and Table 2. The antioxidant activity is expressed in terms of % inhibition and IC_50_ (effective concentration for scavenging 50% of the initial DPPH) value (µM).

Based on the experimental results, among all the compounds synthesized, hydrazinecarbothioamides **4**–**6** showed higher scavenging activity towards DPPH. These compounds have shown a strong inhibitory effect on DPPH radical at 250 μM concentration and inhibition rates were: 97.18% ± 1.42% (for **4**), 96.90% ± 1.39% (for **5**), 97.11% ± 1.12% (for **6**) better than the positive control AA (91.26% ± 0.49%) and BHA (89.30% ± 1.37%) and much stronger than BHT (23.05% ± 1.32%). These compounds **4**–**6** inhibited the DPPH activity with an IC_50_ = 39.39 μM (**4**), 39.79 μM (**5**) and 42.32 μM (**6**) which is better than the specific inhibitor BHA (IC_50_ = 51.62 μM) and AA (IC_50_ = 107.67 μM) and much stronger than BHT (423.37 μM).

The 1,2,4-triazole-3-thiones **7**–**9** obtained by cyclization of hydrazinecarbothioamides showed (at the same concentration, 250 μM) a good antioxidant activity (**7**: 67.70% ± 1.68%, **8**: 72.45% ± 1.42%, **9**: 58.52% ± 1.55%) but lower than AA (91.26% ± 0.49%) and BHA (89.30% ± 1.37%). However, triazoles had higher antioxidant activity than BHT. As deduced from the IC_50_ data, the triazole with the lowest anti-radical capacity were found to be derivative **9** (with 182.60 μM) followed by **7** (147.79 μM) and **8** was found to be slightly more active (133.80 μM) than its counterparts **7** and **9** (Table 1).

The S-alkylated 1,2,4-triazoles **10**–**15** showed weak inhibitory effect at 250 μM concentration, in the range of 7.73%–15.04% (Table 2). However, the presence of the third fluorine atom on phenyl radical linked to ketone groups determines a slight increase of antioxidant activity of compounds **13**–**15** compared with **10**–**12**. Because these compounds presented a weaker action even than BHT, IC_50_ was not calculated.

**Table 1:** Antioxidant activity of compounds **4**–**9** by DPPH method.

Compd.	Scavenging Effect (%)						IC_50_ (μM)
-	25 μM	50 μM	75 μM	100 μM	125 μM	250 μM	-
**4**	30.54 ± 1.32	64.37 ± 1.35	74.86 ± 1.40	85.39 ± 1.45	95.99 ± 1.50	97.18 ± 1.42	39.39
**5**	30.39 ± 1.18	63.58 ± 1.62	74.12 ± 1.34	84.69 ± 1.83	95.36 ± 1.87	96.90 ± 1.39	39.79
**6**	29.14 ± 1.53	59.28 ± 1.23	71.23 ± 1.32	83.23 ± 1.42	95.35 ± 1.18	97.11 ± 1.12	42.32
**7**	15.88 ± 1.03	24.74 ± 1.32	33.30 ± 1.67	37.93 ± 1.49	46.14 ± 1.45	67.70 ± 1.68	147.79
**8**	15.56 ± 0.95	24.36 ± 1.19	32.18 ± 1.48	40.58 ± 1.41	48.38 ± 1.54	72.45 ± 1.42	133.80
**9**	13.96 ± 0.97	22.99 ± 1.05	31.74 ± 1.56	38.63 ± 1.59	43.03 ± 1.63	58.52 ± 1.55	182.60
**AA**	0.70 ± 1.00	1.08 ± 0.84	17.48 ± 1.03	34.91 ± 0.69	84.12 ± 0.48	91.26 ± 0.49	107.67
**BHA**	23.27 ± 1.39	48.99 ± 1.42	64.77 ± 1.32	73.89 ± 1.59	81.74 ± 1.45	89.30 ± 1.37	51.62
**BHT**	-	-	-	-	-	23.05 ± 1.32	423.37

**Table 2:** Antioxidant activity of compounds **10**–**15** by DPPH method.

Compd.	Concentration (μM)	Scavenging Effect (%)
**10**	250	12.67 ± 0.82
**11**	250	8.24 ± 1.20
**12**	250	7.73 ± 0.96
**13**	250	13.23 ± 0.48
**14**	250	15.04 ± 0.43
**15**	250	12.73 ± 0.50
**AA**	250	91.26 ± 0.49
**BHA**	250	89.30 ± 1.37
**BHT**	250	23.05 ± 1.32

**Scheme 2:**
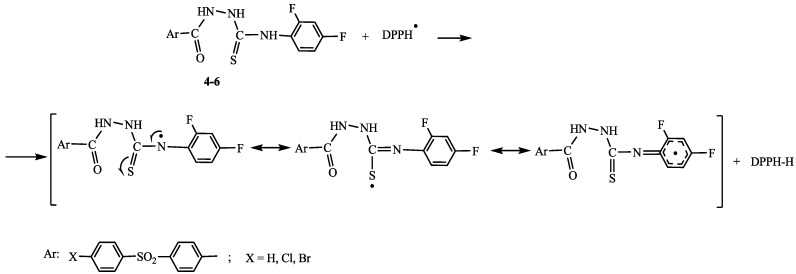
The probable mechanism for the reaction of compounds **4**–**6** with DPPH radical

The higher antioxidant activity of hydrazinecarbothioamides **4**–**6** can be explained by the existence of the thiourea fragment [13] that determines stabilization of free radicals of nitrogen atoms (occurring due to the elimination of hydrogen atoms linked to these) by double conjugation, mainly with the thione group. The conjugation between free radicals of the nitrogen atom and π electrons of the aromatic ring represents an additional factor for increasing the stability of the radical structure. The probable mechanism for the reaction of compounds **4**–**6** with DPPH radical is presented in Scheme 2. 

**Scheme 3:**
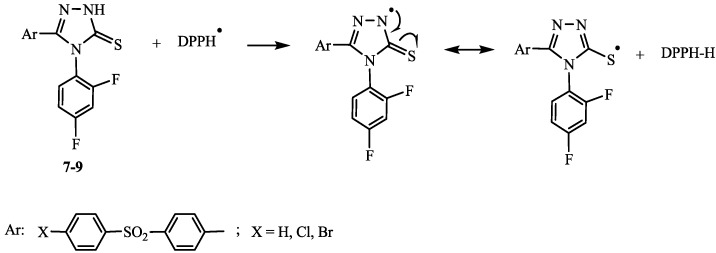
The probable mechanism for the reaction of compounds **7**–**9** with DPPH radical

Heterocyclization to 1,2,4-triazole-3-thiones creates only the possibility to conjugate free radicals on the nitrogen atom N-2 with the thione group, which would explain the lower stability of this radical, probably responsible for a weaker antioxidant activity (Scheme 3).

S-alkylation decreases almost entirely the possibilities of conjugation with thione group, causing the least stable radical structure and the weakest antioxidant activity, according to this interpretation.

The compounds tested displayed a considerable lipophilic character, with estimated mean logP values of 4.65 ± 0.71 (ADMET Predictor, Simulation Plus Inc., Lancaster, CA, USA). Based on the preliminary evaluation of biorelevant molecular descriptors and physico-chemical properties, it appears that the evaluated compounds are typical, low solubility—high permeability entities. Therefore, their bioavailability will dependent on the nature of the administration pathways. For oral route, the solubility in the hydrophilic gastro-intestinal fluids is probably the limiting step for absorption. The *in-vivo* distribution process may include binding and accumulation phenomenon in various organs, likely to be of interest for the antioxidant activity. Moreover, the high lipophilicity can limit the distribution to deeper skin layers or the systemic exposure, which is a considerable advantage for the safety profile. None of the compounds seems to present a high risk of low bioavailability, based on current mnemotic rules [47].

The lipophilicity, as estimate by logP values, was not directly correlated with the antioxidant activity (Supplementary data). Nevertheless, a rank order relationship seems apparent. The compounds showing higher scavenging activity towards DPPH (**4**–**6**) presented the lowest lipophilicity (except for triazole 7 which has lower antioxidant activity than hydrazinecarbothioamides **5** and **6** although it has lower lipophilicity than these derivatives).

## 3. Experimental

### 3.1. Chemistry

All reactants and solvents were obtained commercially with the highest purity and were used without further purification. Melting points were determined on a Boetius apparatus and are uncorrected. The IR spectra were recorded in KBr using a Vertex 70 Bruker spectrometer. Elemental analyses were performed on a ECS-40-10-Costeh micro-dosimeter (and are within ±0.4% of the theoretical values). The NMR spectra were recorded on a Varian Gemini 300 BB instrument operating at 300 MHz for a ^1^H and 75 MHz for ^13^C. Chemical shifts (δ, ppm) were assigned according to the internal standard signal of tetramethylsilane in DMSO-*d*_6_ (δ = 0 ppm). Coupling constants, *J*, are expressed in hertz (Hz). Mass spectra were recorded on 1200 L/MS/MS triple quadrupole (Varian, Palo Alto, CA, USA) spectrometer. In case of compounds **4**–**9**, solutions of 2 μg/mL in methanol/ammonia (1/1, *v*/*v*) were directly injected into the electrospray interface (ESI), after a tenth dilution with methanol, at a flow rate of 20 μL/min. The instrument was operated in positive and negative ions mode. In case of compounds **10**–**15**, methanolic solutions of 0.1 µg/mL (with 0.1% ammonia) were directly infused into APCI (Atmospheric Pressure Chemical Ionization) source with a Prostar 240 SDM Pump (Varian). Parameters for APCI operation were set up as follows: air drying gas at 300 °C and 20 psi, nitrogen as nebulising gas at 40 psi, air as auxiliary gas at 20 psi, APCI torch at 300 °C, and corona discharge needle current at 10 µA. APCI generated only positive ions. Protonated molecular ions were fragmented by collision with argon at 1.5 mTorr.

#### 3.1.1. General Procedure for the Preparation of 2-(4-(4-X-Phenylsulfonyl)benzoyl)-*N*-(2,4-difluorophenyl)hydrazinecarbothioamides **4**–**6**

Appropriate acid hydrazide **1**–**3** (5 mmol) in absolute ethanol (25 mL) and 2,4-difluorophenyl isothiocyanate (5 mmol) was heated under reflux for 10 h. The precipitate formed was cooled, filtered, washed with cold ethanol, dried and recrystallized from ethanol.

*N*-(2,4-Difluorophenyl)-2-(4-(phenylsulfonyl)benzoyl)hydrazinecarbothioamide **4**. Yield: 92.0%; m.p. 176–178 °C; IR (KBr, ν, cm^−1^): 3267, 3169, 3150 (NH), 3067, 3001 (aromatic C-H), 1663 (C=O), 1539, 1510, 1483 (C=C), 1320, 1309, 1155 (SO_2_), 1258 (C=S), 1144 (C-F); ^1^H-NMR (DMSO-*d*_6_, δ ppm) *:* 10.89 (s, 1H; NH); 10.03 (s, 1H, NH); 9.61 (s, 1H, NH); 8.13 (d, 2H, *J* = 8.8 Hz, aromatic protons); 8.09 (d, 2H, *J* = 8.8 Hz; aromatic protons); 7.99 (dd, 2H, *J* = 7.4, 1.4 Hz, aromatic protons); 7.70 (tt, 1H, *J* = 7.4, 1.4 Hz, aromatic proton); 7.63 (t, 2H, *J* = 7.4 Hz, aromatic protons); 7.29 (m, 2H, aromatic protons); 7.07 (wt, 1H, *J* = 8.4 Hz, aromatic proton); ^13^C-NMR (DMSO-*d*_6_, δ ppm): 182.47 (C=S), 164.75 (C=O), 160.57 (dd, *J*_C-F_ = 245.1; 11.2 Hz), 158.55 (dd, *J*_C-F_ = 248.5; 13.7 Hz), 143.84, 140.64, 137.01, 134.12, 131.87 (d, *J*_C-F_ = 9.5 Hz), 129.95, 129.40, 127.57, 127.40, 123.81 (d, *J*_C-F_ = 12.9 Hz), 111.07 (d, *J*_C-F_ = 22.6 Hz), 104.28 (t, *J*_C-F_ = 25.5 Hz); (ESI-MS) *m*/*z*: 448 [M + H]^+^, 319 (38) [C_6_H_5_SO_2_C_6_H_4_CONHNHCS]^+^, 277 (100, BP) [C_6_H_5_SO_2_C_6_H_4_CONHNH_2_ + H]^+^, 245 (19) [C_6_H_5_SO_2_C_6_H_4_CO]^+^; (ESI-MS) *m*/*z*: 446 [M − H]^−^, 426 (2) [M − H-HF]^−^, 412 (11) [M − H-H_2_S]^−^, 275 (100, BP) [C_6_H_5_SO_2_C_6_H_4_CONHNH]^−^; Anal. calcd for C_20_H_15_F_2_N_3_O_3_S_2_ (447.48 g/mol): C, 53.68; H, 3.38; N, 9.39. Found: C, 53.61; H, 3.30; N, 9.28%.

2-(4-(4-Chlorophenylsulfonyl)benzoyl)-*N*-(2,4-difluorophenyl)hydrazinecarbothioamide **5**. Yield: 90%; m.p. 170–172 °C; IR (KBr, ν, cm^−1^): 3290, 3160 (NH), 3090, 3010 (aromatic C-H), 1680 (C=O), 1531, 1478 (C=C), 1319, 1294, 1156 (SO_2_), 1243 (C=S), 1145 (C-F), 761 (C-Cl); ^1^H-NMR (DMSO-*d*_6_, δ ppm) *:* 10.90 (s, 1H, NH); 10.03 (s, 1H, NH); 9.61 (s, 1H, NH); 8.12 (d, 2H, *J* = 8.9 Hz, aromatic protons); 8.09 (d, 2H, *J* = 8.9 Hz, aromatic protons); 8.01 (d, 2H, *J* = 8.5 Hz, aromatic protons); 7.71 (d, 2H, *J* = 8.5 Hz, aromatic protons); 7.07 (wt, 1H, *J* = 8.4 Hz, aromatic protons); 7.29 (m, 2H, aromatic protons); ^13^C-NMR (DMSO-*d*_6_, δ ppm): 182.47 (C=S), 164.71 (C=O), 160.65 (dd, *J*_C-F_ = 246.0; 11.3 Hz), 157.80 (dd, *J*_C-F_ = 245.0; 13.7 Hz), 143.36, 139.45, 138.98, 137.20, 131.82 (d, *J*_C-F_ = 9.7 Hz), 130.11, 129.58, 129.47, 127.54, 123.72 (d, *J*_C-F_ = 19.9 Hz), 111.08 (d, *J*_C-F_ = 21.8 Hz), 104.28 (t, *J*_C-F_ = 25.5 Hz); (ESI-MS) *m*/*z*: 482 [M + H]^+^, 484 [M + H]^+^, 353 (31) [^35^ClC_6_H_4_SO_2_C_6_H_4_CONHNHCS]^+^, 355 (58) [^37^ClC_6_H_4_SO_2_C_6_H_4_CONHNHCS]^+^, 311 (100, BP) [^35^ClC_6_H_4_SO_2_C_6_H_4_CONHNH_2_ + H]^+^, 313 (100, BP) [^37^ClC_6_H_4_SO_2_C_6_H_4_CONHNH_2_ + H]^+^, 279 (5) [^35^ClC_6_H_4_SO_2_C_6_H_4_CO]^+^, 281 (24) [^37^ClC_6_H_4_SO_2_C_6_H_4_CO]^+^; (ESI-MS) *m*/*z*: 480 [M − H]^−^, 482 [M − H]^−^, 446 (9) [^35^ClM-H-H_2_S]^−^, 448 (9) [^37^ClM-H-H_2_S]^−^, 309 (100, BP) [^35^ClC_6_H_4_SO_2_C_6_H_4_CONHNH]^−^, 311 (100, BP) [^37^ClC_6_H_4_SO_2_C_6_H_4_CONHNH]^−^; Anal. calcd for C_20_H_14_ClF_2_N_3_O_3_S_2_ (481.92 g/mol): C, 49.84; H, 2.93; N, 8.72. Found: C, 49.75; H, 2.87; N, 8.60%.

2-(4-(4-Bromophenylsulfonyl)benzoyl)-*N*-(2,4-difluorophenyl)hydrazinecarbothioamide **6**. Yield: 88%; m.p. 175–177 °C; IR (KBr, ν, cm^−1^): 3319, 3280 (NH), 3088, 3044, 3010 (aromatic C-H), 1682 (C=O), 1573, 1536, 1481 (C=C), 1321, 1293, 1157 (SO_2_), 1245 (C=S), 1144 (C-F), 576 (C-Br); ^1^H-NMR (DMSO-*d*_6_, δ ppm) *:* 10.90 (s, 1H, NH); 10.04 (s, 1H, NH); 9.62 (s, 1H, NH); 8.11 (d, 2H, *J* = 8.5 Hz, aromatic protons); 8.10 (d, 2H, *J* = 8.5 Hz, aromatic protons); 7.92 (d, 2H, *J* = 8.7 Hz; aromatic protons); 7.85 (d, 2H, *J* = 8.7 Hz, aromatic protons); 7.29 (m, 2H, aromatic protons); 7.07 (wt, 1H, *J* = 8.8 Hz, aromatic proton); ^13^C-NMR (DMSO-*d*_6_, δ ppm): 182.47 (C=S), 164.71 (C=O), 160.65 (dd, *J*_C-F_ = 243.0; 11.2 Hz), 157.40 (dd, *J*_C-F_ = 243.0; 13.2 Hz), 143.33, 139.86, 137.20, 133.05, 131.93 (d, *J*_C-F_ = 9.6 Hz), 129.60, 129.46, 128.46, 127.54, 123.70, 111.08 (d, *J*_C-F_ = 20.9 Hz), 104.28 (t, *J*_C-F_ = 25.2 Hz); (ESI-MS) *m*/*z*: 526 [M + H]^+^, 528 [M + H]^+^, 397 (40) [^79^BrC_6_H_4_SO_2_C_6_H_4_CONHNHCS]^+^, 399 (33) [^81^BrC_6_H_4_SO_2_C_6_H_4_CONHNHCS]^+^, 355 (100, BP) [^79^BrC_6_H_4_SO_2_C_6_H_4_CONHNH_2_ + H]^+^, 357 (100, BP) [^81^BrC_6_H_4_SO_2_C_6_H_4_CONHNH_2_ + H]^+^, 323 (1) [^79^BrC_6_H_4_SO_2_C_6_H_4_CO]^+^, 325 (32) [^81^BrC_6_H_4_SO_2_C_6_H_4_CO]^+^, 172 (5) [2,4-diFC_6_H_3_NHCS]^+^, 130 (6) [2,4-diFC_6_H_3_NH_2_ + H]^+^; (ESI-MS) *m*/*z*: 524 [M − H]^−^, 526 [M − H]^−^, 504 (3) [M − H-HF]^−^, 506 (4) [M − H-HF]^−^, 490 (7) [M − H-H_2_S]^−^, 492 (11) [M − H-H_2_S]^−^, 353 (100, BP) [^79^BrC_6_H_4_SO_2_C_6_H_4_CONHNH]^−^, 355 (100, BP) [^81^BrC_6_H_4_SO_2_C_6_H_4_CONHNH]^−^; Anal. calcd for C_20_H_14_BrF_2_N_3_O_3_S_2_ (526.37 g/mol): C, 45.64; H, 2.68; N, 7.98. Found: C, 45.58; H, 2.60; N, 7.88%.

#### 3.1.2. General Procedure for the Preparation of 5-(4-(4-X-Phenylsulfonyl)phenyl)-4-(2,4-difluorophenyl)-2H-1,2,4-triazole-3(4H)-thiones **7**–**9**

The corresponding hydrazinecarbothioamide **4**–**6** (3 mmol) was refluxed in aqueous sodium hydroxide solution (8%, 45 mL) for 5 h. The filtrate obtained by filtration of reaction mixture was cooled and acidified to pH~5 with hydrochloric acid (1%). The precipitated obtained was filtered, washed with water, dried and recristallized from CHCl_3_/petroleum ether (1:2, *v*/*v*).

4-(2,4-Difluorophenyl)-5-(4-(phenylsulfonyl)phenyl)-2H-1,2,4-triazole-3(4H)-thione **7** Yield: 71%; m.p. 256–258 °C; IR (KBr, ν, cm^−1^): 3414 (NH), 3065, 3015 (aromatic C-H), 1614, 1580, 1518, 1474 (C=N + C=C), 1338, 1290, 1160 (SO_2_), 1247 (C=S), 1143 (C-F); ^1^H-NMR (DMSO-*d*_6,_ δ ppm): 14.48 (s, 1H, NH); 8.00 (d, 2H, *J* = 8.6 Hz, aromatic protons); 7.95 (dd, 2H, *J* = 7.7, 1.5 Hz, aromatic protons); 7.73 (td, 1H, *J* = 8.7, 6.1 Hz, aromatic proton); 7.70 (t, 1H, *J* = 7.7, 1.5 Hz, aromatic proton); 7.61 (t, 2H, *J* = 7.7 Hz, aromatic protons); 7.58 (d, 2H, *J* = 8.6 Hz, aromatic protons); 7.54 (ddd, 1H, *J* = 10.2, 8.9, 2.7 Hz, aromatic protons); 7.31 (dddd, 1H, *J* = 9.8, 6.1, 2.7, 1.5 Hz, aromatic proton); ^13^C-NMR (DMSO-*d*_6_, δ ppm): 169.52 (C3-triazolic ring), 162.96 (dd, *J*_C-F_ = 251.4; 11.4 Hz), 157.72 (dd, *J*_C-F_ = 252.8; 13.5 Hz), 149.03 (C5-triazolic ring), 142.85, 140.24, 134.17, 132.75 (d, *J*_C-F_ = 10.6, Hz), 129.93, 129.86, 128.80, 128.03, 127.62, 118.39 (d, *J*_C-F_ = 12.7 Hz), 112.99 (d, *J*_C-F_ = 22.9 Hz), 105.68 (t, *J*_C-F_ = 23.8 Hz); (ESI-MS) *m*/*z*: 430 [M + H]^+^; 356 (8) [M + H-SCNNH_2_]^+^; 289 (100, BP) [M + H-C_6_H_5_SO_2_]^+^; 172 (10.9) [F_2_C_6_H_3_NCS + H]^+^; 153 (62) [FC_6_H_4_NCS]^+^; (ESI-MS) *m*/*z*: 428 [M − H]^−^; 408 (15.4) [M − H-HF]^−^; 388 (15.4) [M − H-2HF]^−^; 267 (7,3) [M − H-HF-C_6_H_5_SO_2_]^−^; 141 (100, BP) [C_6_H_5_SO_2_]^−^; Anal. calcd for C_20_H_13_F_2_N_3_O_2_S_2_ (429.46 g/mol): C, 55.93; H, 3.05; N, 9.78. Found: C, 55.83; H, 2.98; N, 9.65%.

5-(4-(4-Chlorophenylsulfonyl)phenyl)-4-(2,4-difluorophenyl)-2H-1,2,4-triazole-3(4H)-thione **8** Yield: 73%; m.p. 245–247 °C; IR (KBr, ν, cm^−1^): 3278 (NH), 3091, 3053 (aromatic C-H), 1614, 1580, 1518, 1468 (C=N + C=C), 1338, 1276, 1159 (SO_2_), 1248 (C=S), 1144 (C-F), 768 (C-Cl); ^1^H-NMR (DMSO-*d*_6_, δ ppm): 14.42 (s, 1H, NH); 8.01 (d, 2H, *J* = 8.5 Hz, aromatic protons); 7.96 (d, 2H, *J* = 8.8 Hz, aromatic proton); 7.73 (td, 1H, *J* = 8.8, 6.0 Hz, aromatic proton); 7.68 (d, 2H, *J* = 8.8 Hz, aromatic protons); 7.59 (d, 2H, *J* = 8.5 Hz, aromatic protons); 7.53 (ddd, 1H, *J* = 10.2, 9.1, 2.7 Hz, aromatic protons); 7.31 (dddd, 1H, *J* = 9.8, 6.4, 2.7, 1.5 Hz, aromatic proton); ^13^C-NMR (DMSO-*d*_6_, δ ppm): 169.54 (C3-triazolic ring), 162.98 (dd, *J* = 250.8; 11.7 Hz), 149.01 (C5-triazolic ring), 157.27 (dd, *J*_C-F_ = 253.1, 13.1 Hz), 142.39, 139.36, 139.04, 132.77 (d, *J*_C-F_ = 10.5 Hz), 130.78, 130.11, 129.63, 128.86, 128.13, 118.40 (d, *J*_C-F_ = 12.6 Hz), 113.01 (d, *J*_C-F_ = 22.6 Hz), 105.70 (t, *J*_C-F_ = 23.5 Hz); (ESI-MS) *m*/*z*: 464 [M + H]^+^; *m*/*z*: 466 [M + H]^+^; 289 (100, BP) [M + H-ClC_6_H_4_SO_2_]^+^; (ESI-MS) *m*/*z*: 462 [M − H]^−^; *m*/*z*: 464 [M − H]^−^; Anal. calcd for C_20_H_12_ClF_2_N_3_O_2_S_2_ (463.91 g/mol): C, 51.78; H, 2.61; N, 9.06. Found: C, 51.89; H, 2.47; N, 8.96%.

5-(4-(4-Bromophenylsulfonyl)phenyl)-4-(2,4-difluorophenyl)-2H-1,2,4-triazole-3(4H)-thione **9** Yield: 81%; m.p. 264–266 °C; IR (KBr, ν, cm^−1^): 3414 (NH), 3095, 3073, 3028 (aromatic C-H), 1614, 1572, 1516, 1471 (C=N + C=C), 1330, 1272, 1169 (SO_2_), 1255 (C=S), 1145 (C-F), 578 (C-Br); ^1^H-NMR (DMSO-*d*_6,_ δ ppm): 14.43 (s, 1H, NH); 8.00 (d, 2H, *J* = 8.5 Hz, aromatic protons); 7.88 (d, 2H, *J* = 8.8 Hz, aromatic protons); 7.83 (d, 2H, *J* = 8.8 Hz, aromatic protons); 7.73 (td, 1H, *J* = 8.8, 6.1 Hz, aromatic protons); 7.59 (d, 2H, *J* = 8.5 Hz, aromatic protons); 7.54 (ddd, 1H, *J* = 10.2, 9.0, 2.9 Hz, aromatic proton); 7.31 (dddd, 1H, *J* = 9.7, 6.5, 2.9, 1.4 Hz, aromatic proton); ^13^C-NMR (DMSO-*d*_6_, δ ppm): 169.54 (C3-triazolic ring), 162.98 (dd, *J*_C-F_ = 251.0, 11.5 Hz), 149.01 (C5-triazolic ring), 157.50 (dd, *J*_C-F_ = 254.0; 13.4 Hz), 142.35, 139.49, 133.06, 132.78 (d, *J*_C-F_ = 10.6, Hz), 130.08, 129.66, 128.88, 128.51, 128.14, 118.48 (d, *J*_C-F_ = 12.6 Hz), 113.03 (d, *J*_C-F_ = 22.9 Hz), 105.66 (t, *J*_C-F_ = 23.5 Hz); (ESI-MS) *m*/*z*: 508 [M + H]^+^; *m*/*z*: 510 [M + H]^+^; 289 (100, BP) [M + H-BrC_6_H_4_SO_2_]^+^; 155 (24.8) [^79^BrC_6_H_4_]^+^; 157 (25.6) [^81^BrC_6_H_4_]^+^; 129 (63.2) [F_2_C_6_H_3_NH_2_]^+^; Anal. calcd for C_20_H_12_BrF_2_N_3_O_2_S_2_ (508.36 g/mol): C, 47.25; H, 2.38; N, 8.27. Found: C, 47.13; H, 2.30; N, 8.13%.

#### 3.1.3. General Procedure for the Preparation of 2-(5-(4-(4-X-Phenylsulfonyl)phenyl)-4-(2,4-difluorophenyl)-4H-1,2,4-triazol-3-ylthio)-1-(phenyl/4-fluorophenyl)ethanones **10**–**15**

To a solution of sodium ethoxide (23 mg of sodium in 10 mL of absolute ethanol) was added the corresponding triazole **7**–**9** (1 mmol). The reaction mixture was stirred at room temperature until a solution was obtained. To this solution was added the corresponding α-halogenated ketone (1 mmol) and stirring was continuated for 10 h. The reaction mixture was poured into ice water and the precipitate was filtered off, washed with water and recristallized from ethanol.

2-(4-(2,4-Difluorophenyl)-5-(4-(phenylsulfonyl)phenyl)-4H-1,2,4-triazol-3-ylthio)-1-phenylethanone **10** Yield: 70%; m.p. 176–178 °C; IR (KBr, ν, cm^−1^): 3070, 3038 (aromatic C-H), 2965, 2922 (CH_2_), 1685 (C=O), 1614, 1598, 1515 (C=N + C=C), 1312, 1291, 1161 (SO_2_), 1146 (C-F); ^1^H-NMR (DMSO-*d*_6,_ δ ppm): 8.03 (dd, 2H, *J* = 7.7, 1.3 Hz, aromatic protons); 8.00 (d, 2H, *J* = 8.6 Hz, aromatic protons); 7.97 (dd, 2H, *J* = 7.7, 1.4 Hz, aromatic proton); 7.87 (dt, 1H, *J* = 8.8, 5.8 Hz, aromatic proton); 7.71 (m, 1H, aromatic proton); 7.65 (t, 2H, *J* = 7.7 Hz, aromatic proton); 7.62 (d, 2H, *J* = 8.6 Hz, aromatic protons); 7.60 (m, 2H, aromatic protons); 7.56 (t, 2H, *J* = 7.7 Hz, aromatic protons); 7.40 (m, 1H, aromatic proton); 5.02 (s, 2H, S-CH_2_-); ^13^C-NMR (DMSO-*d*_6_, *δ* ppm): 192.73 (C=O), 163.34 (dd, *J*_C-F_ = 251.9; 11.7 Hz), 156.66 (dd, *J*_C-F_ = 253.4; 13.8 Hz), 153.11 (C3-triazolic ring), 153.05 (C5-triazolic ring), 142.27, 140.36, 135.14, 134.07, 133.82, 131.59 (d, *J*_C-F_ = 10.9 Hz), 130.66, 129.87, 128.83, 128.41, 128.30, 128.04, 127.54, 117.50 (d, *J*_C-F_ = 12.0 Hz), 113.56 (d, *J*_C-F_ = 25.2 Hz), 106.84 (t, *J*_C-F_ = 23.2 Hz), 40.46 (S-CH_2_-); (APCI-MS) *m*/*z*: 548 [M + H]^+^; 514 (5.6) [M + H-H_2_S]^+^; 430 (15.3) [M + H-C_6_H_5_COCH]^+^; 398 (98.8) [M + H-C_6_H_5_COCHS]^+^; 105 (88.8) [C_6_H_5_CO]^+^; 91 (100, BP) [tropylium]^+^; Anal. calcd for C_28_H_19_F_2_N_3_O_3_S_2_ (547.60 g/mol): C, 61.41; H, 3.50; N, 7.67. Found: C, 61.23; H, 3.29; N, 7.48%.

2-(5-(4-(4-Chlorophenylsulfonyl)phenyl)-4-(2,4-difluorophenyl)-4H-1,2,4-triazol-3-ylthio)-1-phenylethanone **11** Yield: 84%; m.p. 191–193 °C; IR (KBr, ν, cm^−1^): 3084, 3040 (aromatic C-H), 2959, 2921 (CH_2_), 1678 (C=O), 1612, 1598, 1580, 1516 (C=N) + C=C), 1328, 1283, 1161 (SO_2_), 1146 (C-F), 767 (C-Cl); ^1^H-NMR (DMSO-*d*_6,_ δ ppm): 8.03 (dd, 2H, *J* = 7.7, 1.4 Hz, aromatic protons); 8.00 (d, 2H, *J* = 8.8 Hz, aromatic protons); 7.96 (d, 2H, *J* = 8.8 Hz, aromatic protons); 7.70 (d, 2H, *J* = 8.8 Hz, aromatic proton); 7.65 (m, 1H, aromatic proton); 7.63 (d, 2H, *J* = 8.8 Hz, aromatic protons); 7.61 (dt, 1H, *J* = 8.8, 6.0 Hz, aromatic proton); 7.56 (t, 2H, *J* = 7.7 Hz, aromatic protons); 7.40 (ddd, 1H, *J* = 10.2, 9.1, 2.8 Hz, aromatic proton); 7.24 (m, 1H, aromatic proton); 5.02 (s, 2H, S-CH_2_-); ^13^C-NMR (DMSO-*d*_6_, δ ppm): 192.72 (C=O), 163.35 (dd, *J*_C-F_ = 251.9, 11.7 Hz), 156.57 (dd, *J*_C-F_ = 253.4, 13.5 Hz), 153.09 (C3-triazolic ring), 153.06 (C5-triazolic ring), 141.80, 139.22, 139.16, 135.14, 133.82, 131.59 (d, *J*_C-F_ = 10.6 Hz), 130.86, 130.03, 129.55, 128.83, 128.41, 128.34, 128.13, 117.55 (d, *J*_C-F_ = 9.2 Hz), 113.57 (d, *J*_C-F_ = 20.4 Hz), 106.84 (t, *J*_C-F_ = 23.5 Hz), 40.35 (S-CH_2_-); (APCI-MS) *m*/*z*: 582 [M + H]^+^; *m*/*z*: 584 [M + H]^+^; 476 (67.2) [M + H-C_6_H_5_CHO]^+^; 478 (82.3) [M + H-C_6_H_5_CHO]^+^; 464 (28.5) [M + H-C_6_H_5_COCH]^+^; 466 (30.2) [M + H-C_6_H_5_COCH]^+^; 444 (40.1) [M + H-C_6_H_5_COCH-HF]^+^; 446 (41.2) [M + H-C_6_H_5_COCH-HF]^+^; 432 (28.4) [M + H-C_6_H_5_COCHS]^+^; 434 (84.1) [M + H-C_6_H_5_COCHS]^+^; 305 (5.7) [M + H-C_6_H_5_COCHS-F_2_C_6_H_3_NH_2_]^+^; 307 (7.2) [M + H-C_6_H_5_COCHS-F_2_C_6_H_3_NH_2_]^+^; 159 (23.4) [35ClC6H4SO]+; 161 (10.3) [37ClC6H4SO]+; 105 (100, BP) [C_6_H_5_CO]^+^; 91 (95.2) [tropylium]^+^; Anal. calcd for C_28_H_18_ClF_2_N_3_O_3_S_2_ (582.04 g/mol): C, 57.78; H, 3.12; N, 7.22. Found: C, 57.67; H, 3.04; N, 7.07%.

2-(5-(4-(4-Bromophenylsulfonyl)phenyl)-4-(2,4-difluorophenyl)-4H-1,2,4-triazol-3-ylthio)-1-phenylethanone **12** Yield: 82%; m.p. 213–215 °C; IR (KBr, ν, cm^−1^): 3083, 3050 (aromatic C-H), 2960, 2922 (CH_2_), 1703 (C=O), 1615, 1598, 1572, 1517 (C=N + C=C), 1321, 1282, 1160 (SO_2_), 1142 (C-F), 580 (C-Br); ^1^H-NMR (DMSO-*d*_6_, δ ppm): 8.03 (dd, 2H, *J* = 7.7, 1.4 Hz, aromatic protons); 8.00 (d, 2H, *J* = 8.8 Hz, aromatic protons); 7.89 (d, 2H, *J* = 8.8 Hz, aromatic protons); 7.87 (dt, 1H, *J* = 8.8, 5.8 Hz, aromatic proton); 7.83 (d, 2H, *J* = 8.8 Hz, aromatic protons); 7.69 (tt, 1H, *J* = 7.7, 1.4 Hz, aromatic proton); 7.65 (m, 1H, aromatic proton); 7.63 (d, 2H, *J* = 8.8 Hz, aromatic protons); 7.56 (t, 2H, *J* = 7.7 Hz, aromatic protons); 7.39 (m, 1H, aromatic proton); 5.02 (s, 2H, S-CH_2_-); ^13^C-NMR (DMSO-*d*_6_, δ ppm): 192.71 (C=O), 163.26 (dd, *J*_C-F_ = 251.7, 11.7 Hz), 156.65 (dd, *J*_C-F_ = 253.4, 13.5 Hz), 153.11 (C3-triazolic ring), 153.06 (C5-triazolic ring), 141.75, 139.57, 135.13, 133.80, 132.98, 131.59 (d, *J*_C-F_ = 10.6 Hz), 130.86, 129.56, 129.30, 128.83, 128.41, 128.35, 128.13, 117.51 (d, *J*_C-F_ = 12.9 Hz), 113.59 (d, *J*_C-F_ = 22.3 Hz), 106.85 (t, *J*_C-F_ = 23.5 Hz), 40.46 (S-CH_2_-); (APCI-MS) *m*/*z*: 626 [M + H]^+^; *m*/*z*: 628 [M + H]^+^; 476 (31.9) [M + H-C_6_H_5_COCHS]^+^; 478 (26.3) [M + H-C_6_H_5_COCHS]^+^; 434 (12.7) [M + H-C_6_H_5_COCH_2_SNCNH]^+^; 436 (33.2) [M + H-C_6_H_5_COCH_2_SNCNH]^+^; 159 (23.4) [^79^BrC_6_H_4_SO]^+^; 161 (10.3) [^81^BrC_6_H_4_SO]^+^; 105(100, BP) [C_6_H_5_CO]^+^; 91 (95.2) [tropylium]^+^; Anal. calcd for C_28_H_18_BrF_2_N_3_O_3_S_2_ (626.49 g/mol): C, 53.68; H, 2.90; N, 6.71. Found: C, 53.54; H, 2.79; N, 6.62%.

2-(4-(2,4-Difluorophenyl)-5-(4-(phenylsulfonyl)phenyl)-4H-1,2,4-triazol-3-ylthio)-1-(4-fluorophe-nyl)ethanone **13** Yield: 72%; m.p. 152–154 °C; IR (KBr, ν, cm^−1^): 3071, 3035 (aromatic C-H), 2960, 2922 (CH_2_), 1682 (C=O), 1614, 1598, 1515 (C=N + C=C), 1313, 1281, 1161 (SO_2_), 1145 (C-F); ^1^H-NMR (DMSO-*d*_6_, δ ppm): 8.11 (dd, 2H, *J* = 8.8, 5.5 Hz, aromatic protons), 7.99 (d, 2H, *J* = 8.5 Hz, aromatic protons); 7.95 (dd, 2H, *J* = 7.4, 1.5 Hz, aromatic protons); 7.86 (dt, 1H, *J* = 8.8, 5.8 Hz, aromatic proton); 7.71 (tt, 1H, *J* = 7.4, 1.5 Hz, aromatic protons); 7.65 (m, 1H aromatic proton); 7.63 (d, 2H, *J* = 8.5 Hz, aromatic protons); 7.62 (t, 2H, *J* = 7.4 Hz, aromatic protons); 7.39 (t, 2H, *J* = 8.8 Hz, aromatic protons); 7.30 (m, 1H, aromatic proton); 4.99 (s, 2H, S-CH_2_-); ^13^C-NMR (DMSO-*d*_6_, δ ppm): 191.45 (C=O), 165.36 (d, *J*_C-F_ = 252.8 Hz), 163.04 (dd, *J*_C-F_ = 250.5; 11.7 Hz), 156.76 (dd, *J*_C-F_ = 250.9; 13.2 Hz), 153.16 (C3-triazolic ring), 153.01 (C5-triazolic ring), 142.32, 140.38, 134.10, 131.95 (d, *J*_C-F_ = 2.7 Hz), 131.60 (d, *J*_C-F_ = 9.6 Hz), 131.55 (d, *J*_C-F_ = 9.4 Hz), 130.67, 129.90, 128.34, 128.07, 127.57, 117.48 (d, *J*_C-F_ = 9.8 Hz), 115.92 (d, *J*_C-F_ = 21.9 Hz), 113.60 (d, *J*_C-F_ = 22.7 Hz), 106.16 (dd, *J*_C-F_ = 235.0, 27.3 Hz), 40.34 (S-CH_2_-); (APCI-MS) *m*/*z*: 566 [M + H]^+^; 428 (18.9) [M + H-FC_6_H_4_COCH_3_]^+^; 398 (35.3) [M + H-FC_6_H_4_COCHS]^+^; 356 (16.8) [M + H-FC_6_H_4_COCH_2_SNCNH]^+^; 137 (26.5) [FC_6_H_4_COCH_2_]^+^; 123 (82.2) [FC_6_H_4_CO]^+^; 109 (100, BP) [FC_6_H_4_N]^+^; Anal. calcd for C_28_H_18_F_3_N_3_O_3_S_2_ (565.59 g/mol): C, 59.46; H, 3.21; N, 7.43. Found: C, 59.23; H, 3.07; N, 7.26%.

2-(5-(4-(4-Chlorophenylsulfonyl)phenyl)-4-(2,4-difluorophenyl)-4H-1,2,4-triazol-3-ylthio)-1-(4-fluorophenyl)ethanone **14** Yield: 85%; m.p. 226–228 °C; IR (KBr, ν, cm^−1^): 3068, 3030 (aromatic C-H), 2965, 2920 (CH_2_), 1682 (C=O), 1615, 1599, 1514 (C=N + C=C), 1322, 1282, 1158 (SO_2_); 1145 (C-F), 768 (C-Cl); ^1^H-NMR (DMSO-*d*_6,_ δ ppm): 8.11 (dd, 2H, *J* = 8.8, 5.5 Hz, aromatic protons), 8.00 (d, 2H, *J* = 8.5 Hz, aromatic protons), 7.97 (d, 2H, *J* = 8.7 Hz, aromatic protons); 7.86 (dt, 1H, *J* = 8.8, 5.8 Hz, aromatic proton); 7.69 (d, 2H, *J* = 8.7 Hz, aromatic protons); 7.65 (m, 1H, aromatic proton), 7.63 (d, 2H, *J* = 8.5 Hz, aromatic protons); 7.39 (t, 2H, *J* = 8.8 Hz, aromatic protons); 7.38 (m, 1H, aromatic proton); 4.99 (s, 2H, S-CH_2_-); ^13^C-NMR (DMSO-*d*_6_, δ ppm): 191.42 (C=O), 165.34 (d, *J*_C-F_ = 252.5 Hz), 163.18 (dd, *J*_C-F_ = 250.4, 11.8 Hz), 156.72 (dd, *J*_C-F_ = 250.8, 13.1 Hz), 153.09 (C3-triazolic ring), 153.04 (C5-triazolic ring), 141.81, 139.22, 139.16, 131.94 (d, *J*_C-F_ = 2.7 Hz), 131.59 (d, *J*_C-F_ = 9.7 Hz), 131.52 (d, *J*_C-F_ = 9.7 Hz), 130.85, 130.05, 128.55, 128.36, 128.14, 117.64 (d, *J*_C-F_ = 9.8 Hz), 115.89 (d, *J*_C-F_ = 21.9 Hz), 113.57 (d, *J*_C-F_ = 19.6 Hz), 106.20 (dd, *J*_C-F_ = 235.0, 27.3 Hz), 40.33 (S-CH_2_-); (APCI-MS) *m*/*z*: 600 [M + H]^+^; *m*/*z*: 602 [M + H]^+^; 123 (52.2) [FC_6_H_4_CO]^+^; 123 (48.3) [FC_6_H_4_CO]^+^; 109 (100, BP) [FC_6_H_4_N]^+^; Anal. calcd for C_28_H_17_ClF_3_N_3_O_3_S_2_ (600.03 g/mol): C, 56.05; H, 2.86; N, 7.00. Found: C, 55.97; H, 2.76; N, 6.87%.

2-(5-(4-(4-Bromophenylsulfonyl)phenyl)-4-(2,4-difluorophenyl)-4H-1,2,4-triazol-3-ylthio)-1-(4-fluorophenyl)ethanone **15** Yield: 80%; m.p. 228–230 °C; IR (KBr, ν, cm^−1^): 3080, 3067 (aromatic C-H), 2963, 2920 (CH_2_), 1682 (C=O), 1612, 1598, 1574, 1515 (C=N + C=C), 1323, 1282, 1159 (SO_2_), 1144 (C-F), 578 (C-Br); ^1^H-NMR (DMSO-*d*_6,_ δ ppm): 8.11 (dd, 2H, *J* = 8.9, 5.4 Hz, aromatic protons),8.00 (d, 2H, *J* = 8.5 Hz, aromatic protons), 7.89 (d, 2H, *J* = 8.8 Hz, aromatic protons); 7.86 (dt, 1H, *J* = 8.8, 5.8 Hz, aromatic proton); 7.64 (d, 2H, *J* = 8.5 Hz, aromatic protons); 7.83 (d, 2H, *J* = 8.8 Hz, aromatic protons); 7.60 (m, 1H, aromatic proton); 7.38 (t, 2H, *J* = 8.9 Hz, aromatic protons); 7.30 (m, 1H, aromatic proton); 4.99 (s, 2H, S-CH_2_-); ^13^C-NMR (DMSO-*d*_6_, δ ppm): 191.42 (C=O), 165.34 (d, *J*_C-F_ = 252.5 Hz), 163.36 (dd, *J*_C-F_ = 250.2, 11.8 Hz), 156.64 (dd, *J*_C-F_ = 252.0, 13.4 Hz), 153.10 (C3-triazolic ring), 153.05 (C5-triazolic ring), 141.78, 139.58, 132.99, 131.92 (d, *J*_C-F_ = 2.6 Hz), 131.59 (d, *J*_C-F_ = 9.6 Hz), 131.52 (d, *J*_C-F_ = 2.7 Hz), 130.86, 129.57, 129.15, 128.36, 128.13, 117.52 (d, *J*_C-F_ = 9.4 Hz), 115.89 (d, *J*_C-F_ = 21.9 Hz), 113.57 (d, *J*_C-F_ = 20.0 Hz), 106.20 (dd, *J*_C-F_ = 235.0, 27.3 Hz), 40.33 (S-CH_2_-); (APCI-MS) *m*/*z*: 644 [M + H]^+^; *m*/*z*: 646 [M + H]^+^; 476 (31.9) [M + H-FC_6_H_4_COCHS]^+^; 478 (26.3) [M + H-FC_6_H_4_COCHS]^+^; 137 (26.5) [FC_6_H_4_COCH_2_]^+^; 137 (37.2) [FC_6_H_4_COCH_2_]^+^; 123 (100, BP) [FC_6_H_4_CO]^+^; 109 (43.1) [FC_6_H_4_N]^+^; 109 (74.2) [FC_6_H_4_N]^+^; Anal. calcd for C_28_H_17_BrF_3_N_3_O_3_S_2_ (644.48 g/mol): C, 52.18; H, 2.66; N, 6.52. Found: C, 52.07; H, 2.57; N, 6.36%.

### 3.2. Antioxidant Activity

The antioxidant activity of all the synthesized compounds was evaluated by DPPH method [14,38] with some modifications and compared with standards (AA, BHA and BHT).

The 400 μM solution of DPPH (2 mL) in ethanol was added to tested sample solutions (2 mL) of different concentrations (50, 100, 125, 200, 250 and 500 μM) in acetone - ethanol 4:96 *v*/*v*. The samples were kept in the dark at room temperature. After 30 min the absorbance values were measured at 517 nm and were converted into the percentage antioxidant activity (%) using the formula [48]:

% = {1 − [(A_sample_ − A_sampleblank_)/A_control_] × 100 (1)

where A_control_ was the absorbance of DPPH solution without sample, A_sample_ was the absorbance of sample solution with DPPH, A_sampleblank_ was the absorbance of the sample solutions without the DPPH.

All analyses were undertaken on three replicates and the results averaged. The IC_50_ values were calculated by linear regression plots, where the abscissa represented the concentration of tested compound solution (50, 100, 125, 200, 250 and 500 μM) and the ordinate represented the average percent of antioxidant activity from three separate tests. The absorbance was measured on a SPECORD 40 Analytik Jena spectrophotometer.

## 4. Conclusions

New hydrazinecarbothioamides, 1,2,4-triazole-3-thiones and S-alkylated 1,2,4-triazole derivatives were synthesized and characterized by IR, ^1^H-NMR, ^13^C-NMR and mass spectral data. All the synthesized compounds **4**–**15** have been investigated for their antioxidant activity. Some of these compounds were found to be significant scavengers of free radicals. The hydrazinecarbothioamides **4**–**6** showed excellent antioxidant activity, more than the standards. 1,2,4-Triazole-3-thiones showed good antioxidant activity, but lower than the key intermediates from hydrazinecarbothioamide class, unlike S-alkylates derivatives that had very low action. These results obtained by preliminary screening of antioxidant activity suggested that the molecules from hydrazinecarbothioamide class might serve as interesting compounds for the development of new antioxidant agents by synthesis of some new derivatives with this structure.

## Supplementary Files

Supplementary File 1 Supplementary Information (PDF, 563 KB)

## References

[1] Ślusarczyk S., Hajnos M., Skalicka-Woźniak K., Matkowski A. (2009). Antioxidant activity of polyphenols from *Lycopus lucidus* Turcz. Food Chem..

[2] Dakubo G.D. (2010). Mitochondrial Genetics and Cance.

[3] Torreggiani A., Tamba M. (2005). Free radical scavenging and metal chelating activity of some therapeutic heterocyclic agents. Trends Heterocycl. Chem..

[4] Karalı N., Güzel Ӧ., Ӧzsoy N., Ӧzbey S., Salman A. (2010). Synthesis of new spiroindolinones incorporating a benzothiazole moiety as antioxidant agents. Eur. J. Med. Chem..

[5] Patil V.P., Markad V.L., Kodam K.M., Waghmode S.B. (2013). Facile preparation of tetrahydro-5*H*-pyrido[1,2,3-*de*]-1,4-benzoxazines via reductive cyclization of 2-(8-quinolinyloxy)ethanones and their antioxidant activity. Bioorg. Med. Chem. Lett..

[6] Azam F., Kozyrev D., Slutsky V. (2010). Therapeutic Potential of Free Radical Scavengers in Neurological Disorders in Handbook of Free Radicals: Formation, Types and Effects.

[7] Groll A.H., Kolve H. (2004). Antifungal agents: *In vitro* susceptibility testing, pharmacodynamics, and prospects for combination therapy. Eur. J. Clin. Microbiol. Infect. Dis..

[8] Kathiravan M.K., Salake A.B., Chothe A.S., Dudhe P.B., Watode R.P., Mukta M.S., Gadhwe S. (2012). The biology and chemistry of antifungal agents: A review. Bioorg. Med. Chem..

[9] Thompson G.R., Cadena J., Patterson T.F. (2009). Overview of antifungal agents. Clin. Chest Med..

[10] Balfour H.H. (1999). Antiviral drugs. N. Engl. J. Med..

[11] Murthy N., Rao A.R., Sastry G.N. (2004). Aromatase inhibitors: A new paradigm in breast cancer treatment. Curr. Med. Chem. Anticancer Agents.

[12] Koparir M., Orek C., Parlak A.E., Söylemez A., Koparir P., Karatepe M., Dastan S.D. (2013). Synthesis and biological activities of some novel aminomethyl derivatives of 4-substituted-5-(2-thienyl)-2,4-dihydro-3*H*-1,2,4-triazole-3-thiones. Eur. J. Med. Chem..

[13] Yehye W.A., Rahman N.A., Alhadi A.A., Khaledi H., Ng S.W., Ariffin A. (2012). Butylated hydroxytoluene analogs: Synthesis and evaluation of their multipotent antioxidant activities. Molecules.

[14] Kuş C., Ayhan-Kılcıgil G., Özbey S., Kaynak F.B., Kaya M., Çoban T., Can-Eke B. (2008). Synthesis and antioxidant properties of novel *N*-methyl-1,3,4-thiadiazol-2-amine and 4-methyl-2*H*-1,2,4-triazole-3(4*H*)-thione derivatives of benzimidazole class. Bioorg. Med. Chem..

[15] Zoumpoulakis P., Camoutsis C., Pairas G., Soković M., Glamočlija J., Potamitis C., Pitsas A. (2012). Synthesis of novel sulfonamide-1,2,4-triazoles, 1,3,4-thiadiazoles and 1,3,4-oxadiazoles, as potential antibacterial and antifungal agent. Biological evaluation and conformational analysis studies. Bioorg. Med. Chem..

[16] Eswaran S., Adhikari A.V., Shetty N.S. (2009). Synthesis and antimicrobial activities of novel quinoline derivatives carrying 1,2,4-triazole moiety. Eur. J. Med. Chem..

[17] Hassan G.S., El-Messery S.M., Al-Omary F.A.M., Al-Rashood S.T., Shabayek M.I., Abulfadl Y.S., Habib E.-S.E., El-Hallouty S.M., Fayad W., Mohamed K.M. (2013). Nonclassical antifolates, part 4. 5-(2-Aminothiazol-4-yl)-4-phenyl-4H-1,2,4-triazole-3-thiols as a new class of DHFR inhibitors: Synthesis, biological evaluation and molecular modeling study. Eur. J. Med. Chem..

[18] Turan-Zitouni G., Kaplancikli Z.A., Yildiz M.T., Chevallet P., Kaya D. (2005). Synthesis and antimicrobial activity of 4-phenyl/cyclohexyl-5-(1-phenoxyethyl)-3-[N-(2-thiazolyl)acetamido]-thio-4H-1,2,4-triazole derivatives. Eur. J. Med. Chem..

[19] Duran A., Dogan H.N., Rollas S. (2002). Synthesis and preliminary anticancer activity of new 1,4-dihydro-3-(3-hydroxy-2-naphthyl)-4-substituted-5*H*-1,2,4-triazoline-5-thiones. Farmaco.

[20] Idrees G.A., Aly O.M., Abuo-Rahma G.E.D.A.A., Radwan M.F. (2009). Design, synthesis and hypolipidemic activity of novel 2-(naphthalen-2-yloxy)propionic acid derivatives as desmethyl fibrate analogs. Eur. J. Med. Chem..

[21] Özadalı K., Özkanlı F., Jain S., Rao P.P.N., Velázquez-Martínez C.A. (2012). Synthesis and biological evaluation of isoxazolo[4,5-*d*]pyridazin-4-(5*H*)-one analogues as potent anti-inflammatory agents. Bioorg. Med. Chem..

[22] Orek C., Koparir P., Koparir M. (2012). *N*-cyclohexyl-2-[5-(4-pyridyl)-4-(p-tolyl)-4H-1,2,4-triazol-3-ylsulfanyl]-acetamide dihydrate: Synthesis, experimental, theoretical characterization and biological activities. Spectrochim. Acta A.

[23] Navidpour L., Shafaroodi H., Abdi K., Amini M., Ghahremani M.H., Dehpour A.R., Shafiee A. (2006). Design, synthesis, and biological evaluation of substituted 3-alkylthio-4,5-diaryl-4*H*-1,2,4-triazoles as selective COX-2 inhibitors. Bioorg. Med. Chem..

[24] Stefanska J., Szulczyk D., Koziol A.E., Miroslaw B., Kedzierska E., Fidecka S., Busonera B., Sanna G., Giliberti G., La Colla P. (2012). Disubstituted thiourea derivatives and their activity on CNS: Synthesis and biological evaluation. Eur. J. Med. Chem..

[25] Šarkanj B., Molnar M., Čačić M., Gille L. (2013). 4-Methyl-7-hydroxycoumarin antifungal and antioxidant activity enhancement by substitution with thiosemicarbazide and thiazolidinone moieties. Food Chem..

[26] Kuş C., Ayhan-KIlcIgil G., Eke B.C., Işcan M. (2004). Synthesis and antioxidant properties of some novel benzimidazole derivatives on lipid peroxidation in the rat liver. Arch. Pharm. Res..

[27] Shelke S., Mhaske G., Gadakh S., Gill C. (2010). Green synthesis and biological evaluation of some novel azoles as antimicrobial agents. Bioorg. Med. Chem. Lett..

[28] Sriram D., Yogeeswari P., Priya D.Y. (2009). Antimycobacterial activity of novel *N*-(substituted)-2-isonicotinoylhydrazinocarbothioamide endowed with high activity towards isoniazid resistant tuberculosis. Biomed. Pharmacother..

[29] Elslager E.F., Gavrilis Z.B., Phillips A.A., Worth D.F. (1969). Repository drugs. IV., 4',4'''-Sulfonylbisacetanilide (acedapsone, DADDS) and related sulfanilylanilides with prolonged antimalarial and antileprotic action. J. Med. Chem..

[30] McMahon J.B., Gulakowski R.J., Weislow O.S., Schultz R.J., Narayanan V.L., Clanton D.J., Pedemonte R., Wassmundt F.W., Buckheit R.W., Decker W.D. (1993). Diarylsulfones, a new chemical class of nonnucleoside antiviral anhibitors of human immunodeficiency virus Type 1 Reverse Transcriptase. Antimicrob. Agents Chemother..

[31] Saeed A., Shaheen U., Hameed A., Kazmi F. (2010). Synthesis and antimicrobial activity of some novel 2-(substituted fluorobenzoylimino)-3-(substituted fluorophenyl)-4-methyl-1,3-thiazolines. J. Fluorine Chem..

[32] Barbuceanu S.-F., Saramet G., Almajan G.L., Draghici C., Barbuceanu F., Bancescu G.  (2012). New heterocyclic compounds from 1,2,4-triazole and 1,3,4-thiadiazole class bearing diphenylsulfone moieties. Synthesis, characterization and antimicrobial activity evaluation. Eur. J. Med. Chem..

[33] Barbuceanu S.-F., Bancescu G., Saramet G., Barbuceanu F., Draghici C., Radulescu F.S., Ionescu A., Negres S. (2013). Synthesis and biological evaluation of some new *N*^1^-[4-(4-Chlorophenylsulfonyl)benzoyl]-*N*^4^-(aryl)-thiosemicarbazides and products of their cyclization. Heteroat. Chem..

[34] Almajan G.L., Innocenti A., Puccetti L., Manole G., Barbuceanu S., Saramet I., Scozzafava A., Supuran C.T. (2005). Carbonic anhydrase inhibitors. Inhibition of the cytosolic and tumor-associated carbonic anhydrase isozymes I, II, and IX with a series of 1,3,4-thiadiazole- and 1,2,4-triazole-thiols. Bioorg. Med. Chem. Lett..

[35] Socea L.-I., Apostol T.V., Şaramet G., Bărbuceanu Ş.-F., Drăghici C., Dinu M. (2012). Synthesis and root growth activity of some new acetylhydrazinecarbothioamides and 1,2,4-triazoles substituted with 5H-dibenzo[a,d]annulene moiety. J. Serb. Chem. Soc..

[36] Şaramet I., Almăjan G.-L., Barbuceanu Ş., Drăghici C., Banciu M.D. (2005). Synthesis of some substituted aroyl thiosemicarbazides, -mercaptotriazoles and -aminothiadiazoles. Rev. Roum. Chim..

[37] Mavrodin A., Zotta V., Stoenescu V.M., Oteleanu D. (1956). Sulfones. IV. New sulfone-hydrazide derivatives. Pharm. Zentr. Deutsch..

[38] Khan I., Ali S., Hameed S., Rama N.H., Hussain M.T., Wadood A., Uddin R., Ul-Haq Z., Khan A., Ali S. (2010). Synthesis, antioxidant activities and urease inhibition of some new 1,2,4-triazole and 1,3,4-thiadiazole derivatives. Eur. J. Med. Chem..

[39] Kumar H., Javed S.A., Khan S.A., Amir M. (2008). 1,3,4-Oxadiazole/thiadiazole and 1,2,4-triazole derivatives of biphenyl-4-yloxy acetic acid: Synthesis and preliminary evaluation of biological properties. Eur. J. Med. Chem..

[40] Liesen A.P., de Aquino T.M., Carvalho C.S., Lima V.T., de Araújo J.M., de Lima J.G., de Faria A.R., de Melo E.J.T., Alves A.J., Alves E.W. (2010). Synthesis and evaluation ofanti-Toxoplasma gondii and antimicrobial activities of thiosemicarbazides, 4-thiazolidinones and 1,3,4-thiadiazoles. Eur. J. Med. Chem..

[41] Akhtar T., Hameed S., Al-Masoudi N.A., Khan K.M. (2007). Synthesis and anti-HIV activity of new chiral 1,2,4-triazoles and 1,3,4-thiadiazoles. Heteroat. Chem..

[42] Salgın-Gökșen U., Gökhan-Kelekçi N., Göktaș Ö., Köysal Y., Kılıç E., Ișık Ș., Aktay G., Özalp M. (2007). 1-Acylthiosemicarbazides, 1,2,4-triazole-5(4*H*)-thiones, 1,3,4-thiadiazoles and hydrazones containing 5-methyl-2-benzoxazolinones: Synthesis, analgesic-anti-inflammatory and antimicrobial activ. Bioorg. Med. Chem..

[43] Al-Deeb O.A., Al-Omar M.A., El-Brollosy N.R., Habib E.E., Ibrahim T.M., El-Emam A.A. (2006). Synthesis, antimicrobial, and antiinflammatory activities of novel 2-[3-(1-adamantyl)-4-substituted-5-tioxo-1,2,4-triazolin-1-yl]acetic acids, 2-[3-(1-adamantyl)-4-substituted-5-tioxo-1,2,4-triazolin-1-yl]-propionic acids and related derivatives. Arzneim.-Forsch./Drug Res..

[44] Saadeh H.A., Mosleh I.M., Al-Bakri A.G., Mubarak M.S. (2010). Synthesis and antimicrobial activity of new 1,2,4-triazole-3-thiol metronidazole derivatives. Monatsh. Chem..

[45] Kumar A., Sharma P., Kumari P., Kalal B.L. (2011). Exploration of antimicrobial and antioxidant potential of newly synthesized 2,3-disubstituted quinazoline-4(3H)-ones. Bioorg. Med. Chem. Lett..

[46] Zhou B., Li B., Yi W., Bu X., Ma L. (2013). Synthesis, antioxidant, and antimicrobial evaluation of some 2-arylbenzimidazole derivatives. Bioorg. Med. Chem. Lett..

[47] Lipinski C.A., Lombardo F., Dominy B.W., Feeney P.J. (2001). Experimental and computational approaches to estimate solubility and permeability in drug discovery and development settings. Adv. Drug. Deliv. Rev..

[48] Duan X.-J., Zhang W.-W., Li X.-M., Wang B.-G. (2006). Evaluation of antioxidant property of extract and fractions obtained from a red alga, Polysiphonia urceolata. Food Chem..

