# Supplementary material for: Synthesis and Antioxidant Activity Evaluation of New Compounds from Hydrazinecarbothioamide and 1,2,4-Triazole Class Containing Diarylsulfone and 2,4-Difluorophenyl Moieties
Source: Int J Mol Sci. 2014 Jun 17;15(6):10908–25. doi: 10.3390/ijms150610908 (PMC4100188; doi:10.3390/ijms150610908)
Supplement: Supplementary File 1 — Supplementary Information (PDF, 563 KB) [file ijms-15-10908-s001.pdf]

Supplementary Information

Table S1. The estimated values of logP and *in silico* permeability across model interfaces.

Name	logP	Human Jejunal Effective Permeability (cm/s × 10^4)	Permeability (MDCK) (cm/s × 10^7)	Permeability (Rabbit Cornea) (cm/s × 10^7)	Permeability (Skin) (cm/s × 10^7)
**4**	3.46	3.34	71.79	71.94	0.17
**5**	4.01	3.57	59.96	87.65	0.05
**6**	4.13	3.75	52	84.62	0.05
**7**	3.82	4.47	88.73	204.61	42.42
**8**	4.42	5.07	89.45	238.88	37.93
**9**	4.52	5.37	74.74	233.1	45.62
**10**	4.71	5.04	236.57	208	1.69
**11**	5.27	5.64	249.17	280.16	15.74
**12**	5.4	5.94	230.41	249.71	15.02
**13**	4.95	5.19	290.51	208.37	7.16
**14**	5.5	5.78	306.07	262.44	64.34
**15**	5.63	6.08	287.23	243.42	67.2

Table S2. The estimated solubility in water and biorelevant gastro-intestinal fluids.

Name	Solubility Water mg/mL	Intrinsic Solubility mg/mL	Solubility at pH = 7.4 mg/mL	Solubility in FaSSGF mg/mL	Solubility in FaSSIF mg/mL	Solubility in FeSSIF mg/mL
**4**	0.00128	0.00116	0.00154	0.01030	0.03820	0.09760
**5**	0.00066	0.00058	0.00075	0.00546	0.02320	0.06990
**6**	0.00079	0.00071	0.00087	0.00475	0.02100	0.06440
**7**	0.00138	0.00115	0.00239	0.01400	0.30900	0.09010
**8**	0.00070	0.00051	0.00138	0.00579	0.17400	0.06340
**9**	0.00080	0.00060	0.00144	0.00495	0.15600	0.05630
**10**	0.00037	0.00037	0.00037	0.00721	0.01800	0.01730
**11**	0.00019	0.00019	0.00019	0.00360	0.01120	0.01230
**12**	0.00026	0.00026	0.00026	0.00308	0.01020	0.01140
**13**	0.00039	0.00039	0.00039	0.00535	0.01350	0.01410
**14**	0.00021	0.00021	0.00021	0.00275	0.00874	0.01020
**15**	0.00028	0.00028	0.00028	0.00234	0.00797	0.00942

FaSSGF—Fast State Simulated Gastric Fluid; FaSSIF—Fast State Simulated Intestinal Fluid; FeSSIF—Fed State Simulated Intestinal Fluid.

Portions of these results were generated by ADMET Predictor™ software provided by Simulations Plus, Inc., Lancaster, CA, USA.

© 2014 by the authors; licensee MDPI, Basel, Switzerland. This article is an open access article distributed under the terms and conditions of the Creative Commons Attribution license (http://creativecommons.org/licenses/by/3.0/).
